# Higher Throughput Quantification of Neutralizing Antibody to Herpes Simplex Viruses

**DOI:** 10.1371/journal.pone.0144738

**Published:** 2015-12-14

**Authors:** Tamara P. Blevins, Michelle C. Mitchell, Maria Korom, Hong Wang, Yinyi Yu, Lynda A. Morrison, Robert B. Belshe

**Affiliations:** 1 Department of Internal Medicine, Division of Infectious Diseases, Saint Louis University School of Medicine, St. Louis, Missouri, United States of America; 2 Department of Molecular Microbiology and Immunology, Saint Louis University School of Medicine, St. Louis, Missouri, United States of America; Cincinnati Childrens Hospital Medical Center, UNITED STATES

## Abstract

We report a rapid, higher throughput method for measuring neutralizing antibody to herpes simplex virus (HSV) in human sera. Clinical isolates and sera from the Herpevac Trial for Women were used in a colorimetric assay in which infection of tissue culture (lack of neutralization) was indicated by substrate metabolism by beta-galactosidase induced in the ELVIS cell line. The neutralization assay was optimized by addition of guinea pig complement, which particularly enhanced neutralizing antibody titers to HSV-2. Higher neutralizing antibody titers were also achieved using virus particles isolated from the supernatant of infected cells rather than lysate of infected cells as the source of virus. The effect of assay incubation time and incubation time with substrate were also optimized. We found that incubating with substrate until a standard optical density of 1.0 was reached permitted a better comparison among virus isolates, and achieved reliable measurement of neutralizing antibody activity. Interestingly, in contrast to results in the absence of complement, addition of complement allowed sera from HSV-2 gD-vaccinated subjects to neutralize HSV-1 and HSV-2 clinical and laboratory isolates with equal potency.

## Introduction

An important aspect in development and testing of vaccines is identification and monitoring of immune responses that correlate with protection. These correlates of protection may consist of several forms of adaptive immunity, including cellular and humoral immune components. Humoral immunity is often assessed by enzyme-linked assays which quantify antibodies in vaccine recipients’ sera capable of specifically binding vaccine antigens. Such assays are simple, inexpensive, high throughput, and can be modified to facilitate identification of individual epitopes and also isotypes and subclasses of antibodies elicited by the vaccine. These assays are not, however, a measure of functional antibody that could be an indicator of efficacy. Neutralizing antibodies identify interactions between the target microbe and immune effectors that reduce infectivity.

Assays of neutralizing activity can be cumbersome and difficult to translate results obtained from one study or study site to the next. Several methods for determining HSV neutralizing antibody titer have been developed including cytopathic effect (CPE) inhibition, plaque reduction, and dye exclusion assays, but each has some limitations. First, all three methods are laborious and entail long assay run times, which from setup to completion can require 4 to 5 days. Second, CPE inhibition readouts are subjective, consisting of infecting cells in the presence of serum for 72 h, then staining and visualizing intact monolayers [1,2], or scoring the monolayer directly for CPE without staining [3]. The plaque reduction method requires a 72 h incubation, and significant labor involved in counting plaques [4,5]. The dye exclusion method entails the same setup as the plaque reduction method but requires a 96 h incubation before cell monolayers are stained and dye uptake is quantified by spectrophotometer [6,7]. An optimized and standardized, higher throughput method of determining neutralizing antibody titers that can be used to directly compare responses to various virus isolates is needed, particularly for evaluating samples from clinical trials of vaccine candidates.

A large phase III field trial of a herpes simplex vaccine consisting of glycoprotein D (gD) in adjuvant (the Herpevac Trial for Women) was conducted in 8,323 young adult women who were seronegative for both HSV-1 and HSV-2 [6]. The vaccine provided 82% protection versus HSV-1 culture positive disease but no protection versus HSV-2. In order to better understand the unexpected results of the Herpevac Trial, we plan to evaluate neutralizing antibody as a correlate of protection. ELISA studies of antibody found that higher titers of binding antibody to gD correlated with protection against HSV-1 [8]. No correlate was identified for HSV-2 but we propose to revisit this question with neutralizing antibody because there was a trend toward reduced HSV-2 infection (p value not significant) among those subjects with higher ELISA titers. Studying clinical isolates from the trial seems most appropriate, because breakthrough disease included culture positive genital disease among the clinical trial endpoints. We therefore developed a higher throughput neutralizing antibody assay that measured neutralization of HSV-1 and HSV-2 strains isolated during the trial, using a quantifiable automatic readout in ELVIS (Enzyme Linked Virus Inducible System) cells [9]. We report here on the method and its characteristics evaluating 8 strains each of HSV-1 and HSV-2 using pre- and post-vaccine sera.

## Materials and Methods

### Sera and reagents

Reference serum consisting of a pool of HSV-1-reactive, defibrinated plasma donations was obtained from the *National Institute for Biological Standards and Control* (Health Protection Agency) (nibsc.org/). Test sera were banked from the Herpevac Trial for Women [6] and include pre- and 7 mo. post-vaccination samples from individual subjects. All subjects were seronegative for HSV-1 and HSV-2 prior to entry into the clinical trial. The study was approved by the Saint Louis University Institutional Review Board (IRB number 24706) and subjects provided written consent to future use of their samples.

Lyophilized guinea pig serum with defined complement activity was obtained from Sigma-Aldrich (St. Louis, MO), Millipore, Quidel Corporation (San Diego, CA), and Cedarlane (Burlington, NC). Human serum as a source of complement was derived from HSV-1/HSV-2-seronegative donors identified using the HerpeSelect assay (Focus Diagnostics, Cypress, CA) also used to screen potential subjects in the Herpevac Trial for Women.

### Cells and Viruses

Baby hamster kidney (BHK) cells stably transformed with a construct containing the HSV-1 ICP6 promoter driving the *E*. coli *lacZ* gene (ELVIS cells), were obtained from Quidel. ELVIS cells were propagated in Eagle’s Minimal Essential medium (EMEM) (Lonza) supplemented with 10% fetal bovine serum (FBS) (Atlanta Biologicals), 2 mM L-glutamine, and 100 IU/ml penicillin/0.1 mg/ml streptomycin (P/S). G418 (Geneticin; Sigma-Aldrich) at 1 mg/ml was added to the ELVIS cell medium every fifth passage to maintain a homogenous beta-galactosidase (β-gal)-expressing cell line. Vero cells were propagated in Dulbecco’s MEM supplemented with 3% bovine growth serum (Hyclone), 3% newborn calf serum (Sigma Aldrich), 2 mM L-glutamine, and 100 IU/ml P/S.

The HSV-1 reference strain KOS [10] and the HSV-2 reference strains G [11] and 333 [12] were grown in Vero cells and cell lysate stocks were prepared as previously described [3]. Primary clinical isolates obtained from swabs during the Herpevac Trial were inoculated into T75 flasks of Vero cells and collected when CPE reached 100%. The infected cells were sonicated and aliquoted to serve as the primary stock. Virus titer was determined from an aliquot of each by standard plaque assay on Vero cells [13]. Virus for neutralization assays was produced free of cell debris by isolation from the supernatant of infected cell monolayers using high-speed centrifugation, as previously described [3]. Briefly, fresh Vero cell monolayers were inoculated at multiplicity of infection (MOI) 0.01 for HSV-1 isolates or 0.1 for HSV-2 isolates, and supernatants were collected when CPE reached 100%. After centrifugation at 300 x g for 10 min to remove detached cells and debris, virions in the clarified supernatants were isolated by centrifugation at 27,000 x g for 60 min at 4°C. Pellets were resuspended in PBS, sonicated, aliquoted, and stored at -70°C. Virus titers were determined by plaque assay on Vero and BHK cells, with very little difference noted.

### Neutralization Assay

The HSV neutralization assay was based on a previously described protocol [14,15]. Individual sera from the Herpevac Trial containing HSV neutralizing antibody activity were used to optimize a colorimetric assay employing the ELVIS cell line. ELVIS cells in 150 cm^2^ flasks were trypsinized (Millipore) and seeded at 3.5x10^4^ cells/ml (3.5x10^3^ cells/well) into 96 well flat-bottomed plates (Costar) and incubated overnight to achieve confluent monolayers. Test sera were heat inactivated for 30 min at 56°C, then serially diluted in two-fold steps in a 96-well U-bottom plate (Corning) from 1:20 to 1:10,240 in 100 μl of EMEM supplemented with 2% FBS and 10% guinea pig serum as a source of complement. Test serum dilutions (100 μl) were combined with 100 μl of HSV-1 or HSV-2 at 1x10^4^ plaque forming units (pfu)/ml and the virus/serum mixtures were incubated for 1 h at 37°C and 5% CO_2_ with gentle rocking. After the 1 h incubation, medium was decanted from the confluent ELVIS plates and 50 μl of the virus/serum mixture was added directly onto each cell monolayer. Virus control wells (no serum) and uninfected control wells (no virus) were included for each sample on the plate. After the virus/serum addition, the plates were incubated at 37°C, 5% CO_2,_ for an additional hour. An additional 100 μl/well EMEM containing 2% FBS was added and the plates were incubated overnight (18 h) at 37μC and 5% CO_2._ After overnight incubation, the medium was carefully aspirated and 50 μl of 1.5% NP40 (Thermo Scientific) in EMEM was added to each well, and the plates were frozen at -70°C. After a minimum of 4 h, the plates were thawed at room temperature (~30 min), and 50 μl/well of substrate [1% Chlorophenolred-β-galactopyranoside (CPRG, Roche Applied Science) prepared in a 2X working buffer (10 mM MgSO_4_, 100 mM KCl, 400 mM Na_2_H_2_PO_4_-H_2_O, and 600 mM Na_2_HPO_4_-7H_2_O)] was added. Plates were incubated at 37°C and 5% CO_2_ until an OD reading of 1.0 was achieved when read at wavelength 570 nm (OD_570_) in a microplate reader (Tecan Sunrise). The appropriate incubation time for each isolate was previously determined in an assay without added patient serum, with periodic readings until the standard OD reading of 1.0 was achieved. Thus, assays with test sera could be read at a single time point for titer determination.

IC_50_ values were calculated by non-linear regression using a 4-parameter logistic dose response curve (GraphPad Prism). Briefly, the OD_570_ was plotted against the log serum dilution to generate a sigmoidal curve. Positive (no serum) and negative (no virus) controls were used to define the constraints for the top and bottom of the curve, respectively, and the 50% neutralization titer was determined mathematically.

### Particle Counts

Virus particle counts were determined by electron microscopy in comparison to latex beads of known concentration. Virus preparations were fixed in 0.25% glutaraldehyde, and mixed in equal parts with latex beads in BSA and with BSA buffer. Preparations were placed on formvar-coated grids and fluid drawn off. The grids were negatively stained with phosphotungstic acid and random images were taken at 15000x on a JEOL 1200x TEM equipped with a side mount AMT digital camera. Fifty images per isolate were counted. A ratio between virus particle and latex bead counts was obtained. The virus particle count was then calculated using this ratio and the concentration of the beads.

### RNA isolation and quantitative real-time PCR

Duplicate cultures of ELVIS cells (2.13 x 10^6^ cells in 6-well plate format), were mock infected or infected at an MOI of 1 with HSV-1 clinical isolates. Total RNA was isolated at 3 h and 6 h post-infection using a PureLink RNA Mini Kit (Life Technologies), followed by a DNase digestion step using a TURBO DNA-free Kit (Life Technologies). Four hundred nanograms of each RNA sample was reverse transcribed using the combination of random hexamers, anchored-oligo(dT)_18_ primers and a Transcriptor First-strand cDNA Synthesis kit (Roche) in a 20-μl volume according to the manufacturer's instructions. Quantitative real-time PCRs to detect ICP0 mRNA and 18S rRNA were performed on cDNA using FastStart Universal Probe Master [Rox] and FastStart SYBR Green Master Mix [Rox] (Roche) respectively on a 7500 Fast Real-Time PCR System (Applied Biosystems). Reactions were performed in triplicate in 25-μl volumes. The primer sequences and the hydrolysis probe for HSV-1 ICP0 mRNA were designed using the Roche Diagnostics' Probe Finder program version 2.50. The ICP0 primers used were ICP0-FWD 5′- ACCACCATGACGACGACTC -3′, ICP0-REV 5′-AGCCCCGTCTCGAACAGT -3′ and Universal ProbeLibrary Probe #56 FAM-5'-GGACAGCA-3'-Dark quencher (Roche), which amplify a 67-bp product. The sequences of the 18S rRNA primers used were 5′-GTAACCCGTTGAACCCCATT-3′ and 5′-CCATCCAATCGGTAGTAGCG-3′, which amplify a 151-bp product. The PCR parameters were 10 min of activation at 95°C, followed by 40 cycles at 95°C for 15 sec and at 60°C for 30 sec. Specificity of the 18S rRNA reaction was verified by melting curve analysis. The ICP0 signal was normalized to the 18S rRNA signal. The signal of clinical isolate #2 at 3 h post-infection was set to 1, and the relative amounts of ICP0 were calculated using the 2^−ΔΔCT^ method [16,17].

## Results

### Neutralizing antibody assay optimization

**Fig 1:**
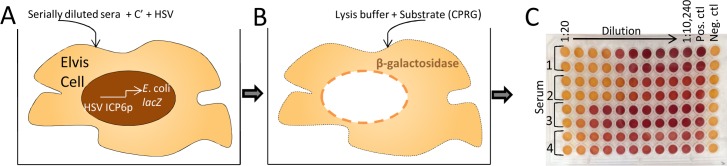
Depiction of the neutralizing antibody assay. Individual sera were evaluated for HSV neutralizing antibody titer in a colorimetric assay employing the ELVIS cell line, a recombinant BHK cell line stably transfected with a construct containing the *E*. *coli lacZ* gene driven by the HSV ICP6 promoter. Heat inactivated, diluted test sera were combined with virus and complement. A) After a 1 h incubation of the virus/serum mixture, 0.05 ml portions were added to ELVIS cell monolayers. Following a 1 h adsorption period, feeding medium was added and plates were incubated. B) After 18 h incubation, culture fluid was aspirated, lysis buffer was added, and plates were frozen. C) Upon thawing, CPRG substrate was added, the plates were incubated, and then absorbance was determined at OD_570_ in a microplate reader_._

A neutralizing antibody assay using ELVIS cells (Fig 1) was previously described and modified [14,15]. We tested various independent variables in this assay to optimize HSV neutralizing antibody detection for reproducible, higher throughput screening of human test sera. The ELVIS cell line used in this assay is stably transfected with the *E*. coli *lacZ* gene under the control of the HSV ICP6 promoter. In the presence of HSV, the *lacZ* gene is transcribed and β-gal is expressed. The level of β-gal expression is detected by metabolism of the CPRG substrate.

To determine the optimal time for exposure of cells to mixtures of virus and subject sera, a time course was performed. Mixtures of virus with two different sera were incubated with cells for 6 h, 9 h, 18 h, or 24 h. The 6 h and 9 h incubations of cells with the virus/serum mixtures produced very low OD readings and low neutralizing antibody titers when compared with the 18 h and 24 h incubations (data not shown). This result indicated that little β-gal had been expressed at these earlier times, which prevented detection of neutralizing activity. Incubation of cells with the virus/serum mixtures for 18 h yielded higher OD readings and higher neutralizing antibody titers. Titers measured after 24 h incubation were similar to those after incubation for 18 h, indicating peak neutralization had occurred by approximately 18 h after addition of the virus/serum mixtures to the cells. Because the OD readings obtained after 24 h incubation were nearer saturation level for both virus/serum mixtures tested, an 18 h incubation period was selected for the assay.

**Fig 2:**
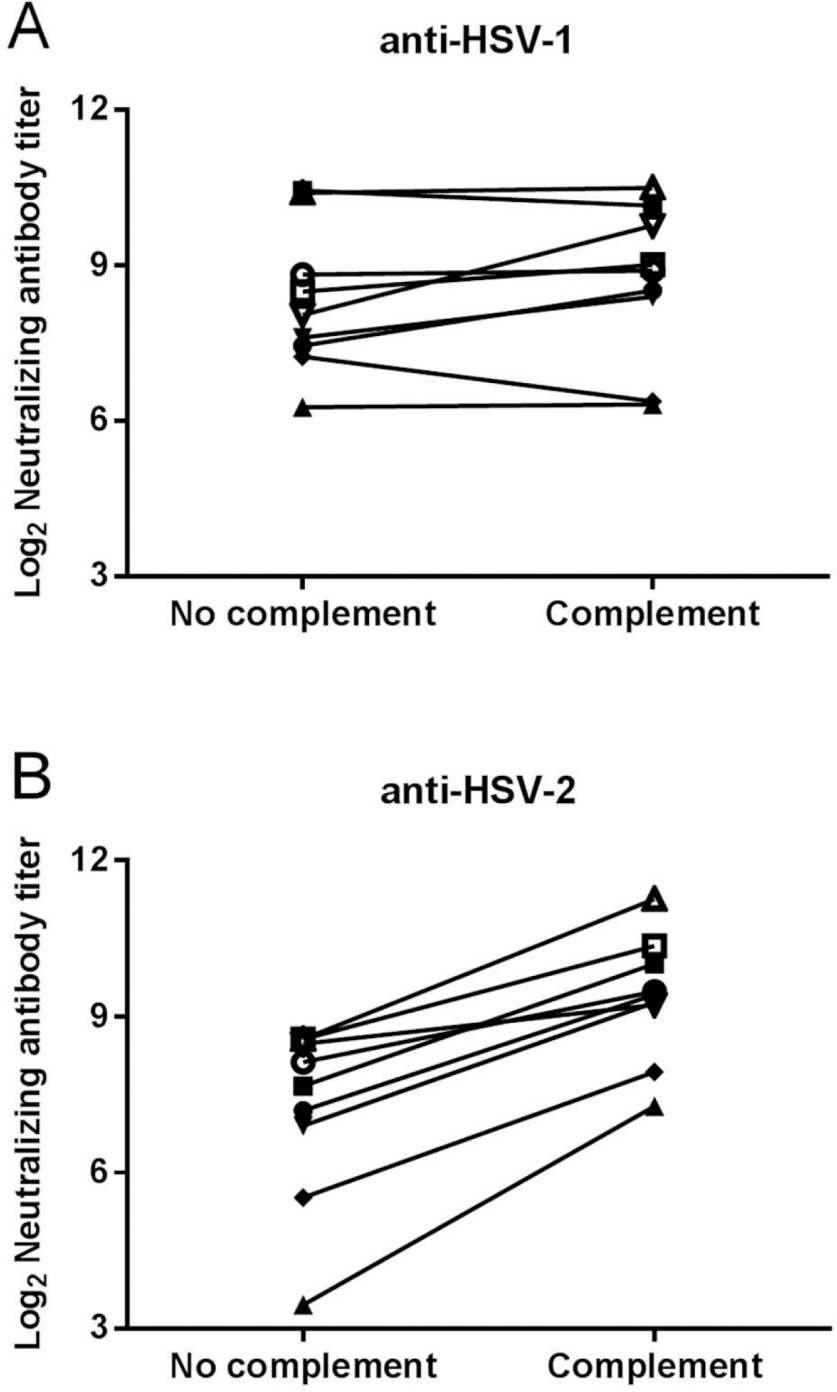
The effect of complement addition to the virus/serum mixture. Virus isolates were prepared in a solution of EMEM containing 2% FBS and 10% guinea pig complement or no complement. The prepared virus was then mixed with test serum from eight different subjects and added to cell monolayers. Neutralizing antibody titers against A) a HSV-1 isolate or B) a HSV-2 isolate were determined as described in Fig 1. HSV-1 without complement versus with complement, P = 0.2071; HSV-2 without complement versus with complement, P = <0.0001 by paired t test.

To determine whether addition of complement significantly affects neutralizing antibody titer, HSV-1 and HSV-2 isolates were mixed with subjects’ sera and incubated in the presence of reconstituted guinea pig serum as a complement source. Without the addition of complement, the mean neutralizing antibody titers against HSV-1 and HSV-2 were 491 and 214, respectively. In contrast, with the addition of complement the mean titers were 591 and 870, respectively (Fig 2). The mean of the fold differences in neutralizing antibody titers for individual sera with the addition of complement thus increased only 1.5-fold for HSV-1 (P = 0.2071), but was significantly (5.4-fold) higher for HSV-2 (P = <0.0001). Guinea pig complement from several commercial sources yielded similar results (data not shown). Quidel complement was selected for the assay because it was available in larger volumes and arrived reconstituted.

Because HSV gC interferes with complement activation [18,19], we compared guinea pig complement to human serum from HSV-1/HSV-2 seronegative donors. Serum from two individual donors was added to the assay medium at concentrations of 7.5% or 10% and compared with standard 10% guinea pig serum. Both donors’ serum yielded neutralizing titers against HSV-1 equal to or greater than guinea pig serum (Table 1). Interestingly, 7.5% human serum generated slightly higher neutralizing titers than addition of 10% human serum. Because addition of guinea pig serum as a source of complement yielded equivalent results to HSV antibody-negative human serum and is commercially available, guinea pig serum was used in further assay development.

**Table 1:** Comparison of human and guinea pig complement (C’) activity.

**HSV-1 Neutralizing Titers**			
**Subject**	**7.5% Human C’**	**10% Human C’**	**10% Guinea pig C’**
1	176	122	
2	367	249	
Avg.	272	186	170
**HSV-2 Neutralizing Titers**			
**Subject**	**7.5% Human C’**	**10% Human C’**	**10% Guinea pig C’**
1	286	223	
2	365	419	
Avg.	326	321	329

To determine the optimal time complement should be added to the assay, two methods of addition were compared. In the first method, 10% guinea pig complement was added to the assay medium. This medium was used to prepare the virus and also the serum dilutions. In the second method, the virus/serum mixtures were prepared without complement and then 20 μl of complement were added to the 96 well plate, resulting in reduced exposure time to complement. These methods of addition yielded comparable results for neutralization of both HSV-1 and HSV-2 isolates by a variety of sera (data not shown), so we opted to add complement to the assay medium for ease of use.

**Fig 3:**
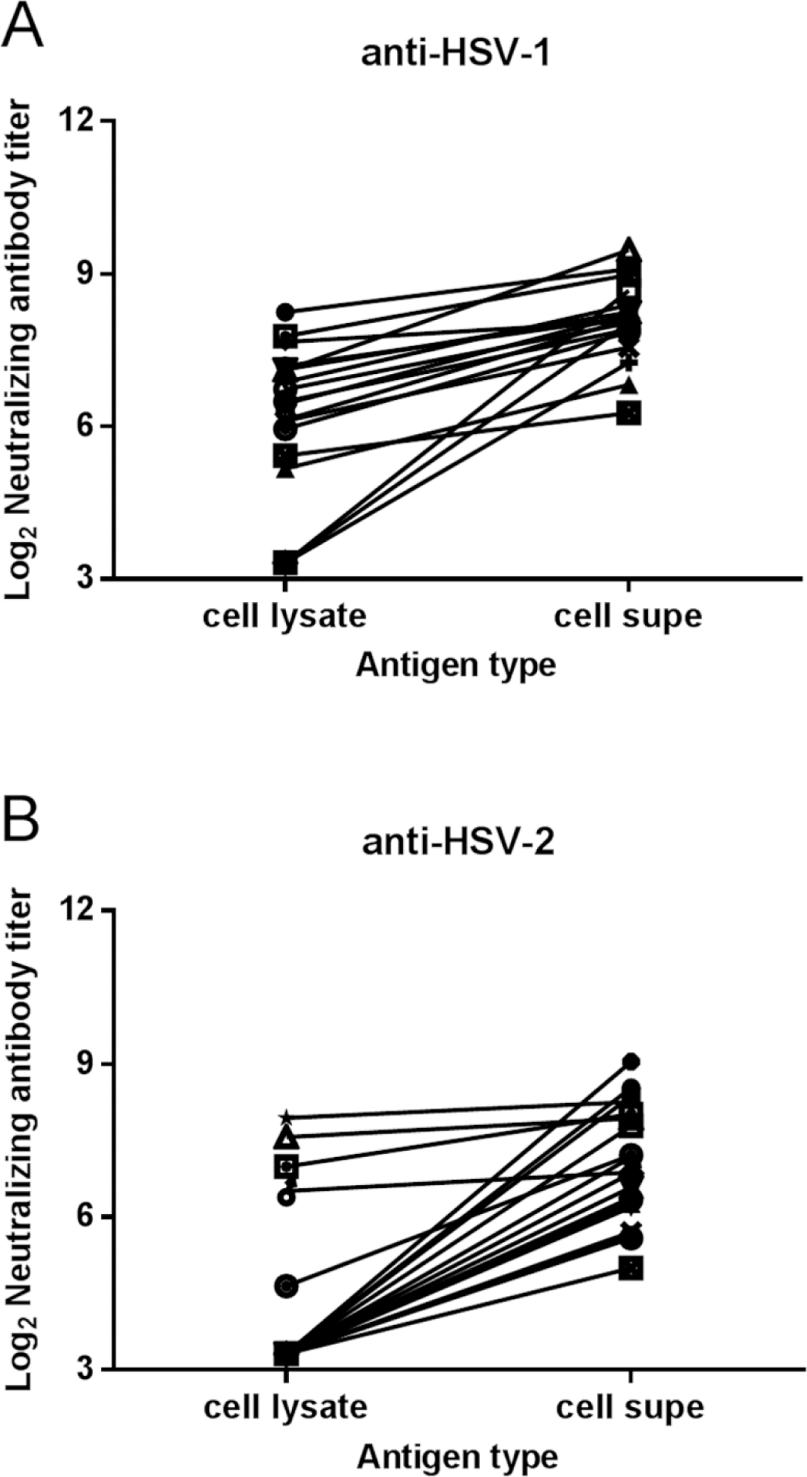
Extracellular virus stocks yield higher neutralizing antibody titers. Equivalent pfu of cell lysate stocks and extracellular virus stocks of the same virus isolates were used in the neutralization assay with various test sera. Neutralizing antibody titers against A) HSV-1 stocks and B) HSV-2 stocks of primary isolates from the Herpevac Trial are shown with various symbols. P < 0.0001 for HSV-1 cell lysate compared with extracellular virus; P < 0.0001 for HSV-2 cell lysate compared with extracellular virus by paired t test.

The effect of using infected cell lysate as the virus source was compared to extracellular virus in the neutralization assay. A cell lysate stock and an extracellular virus stock of several HSV-1 and HSV-2 laboratory strains and primary isolates were prepared. Equivalent pfu of the two types of virus stock mixed with test serum were used to infect ELVIS cells. The mixtures containing extracellular virus consistently produced higher neutralizing antibody titers than the cell lysate virus (Fig 3). Therefore, extracellular virus stock was selected for use in the neutralization assay.

### Variation in β-gal induction by different clinical isolates

**Fig 4:**
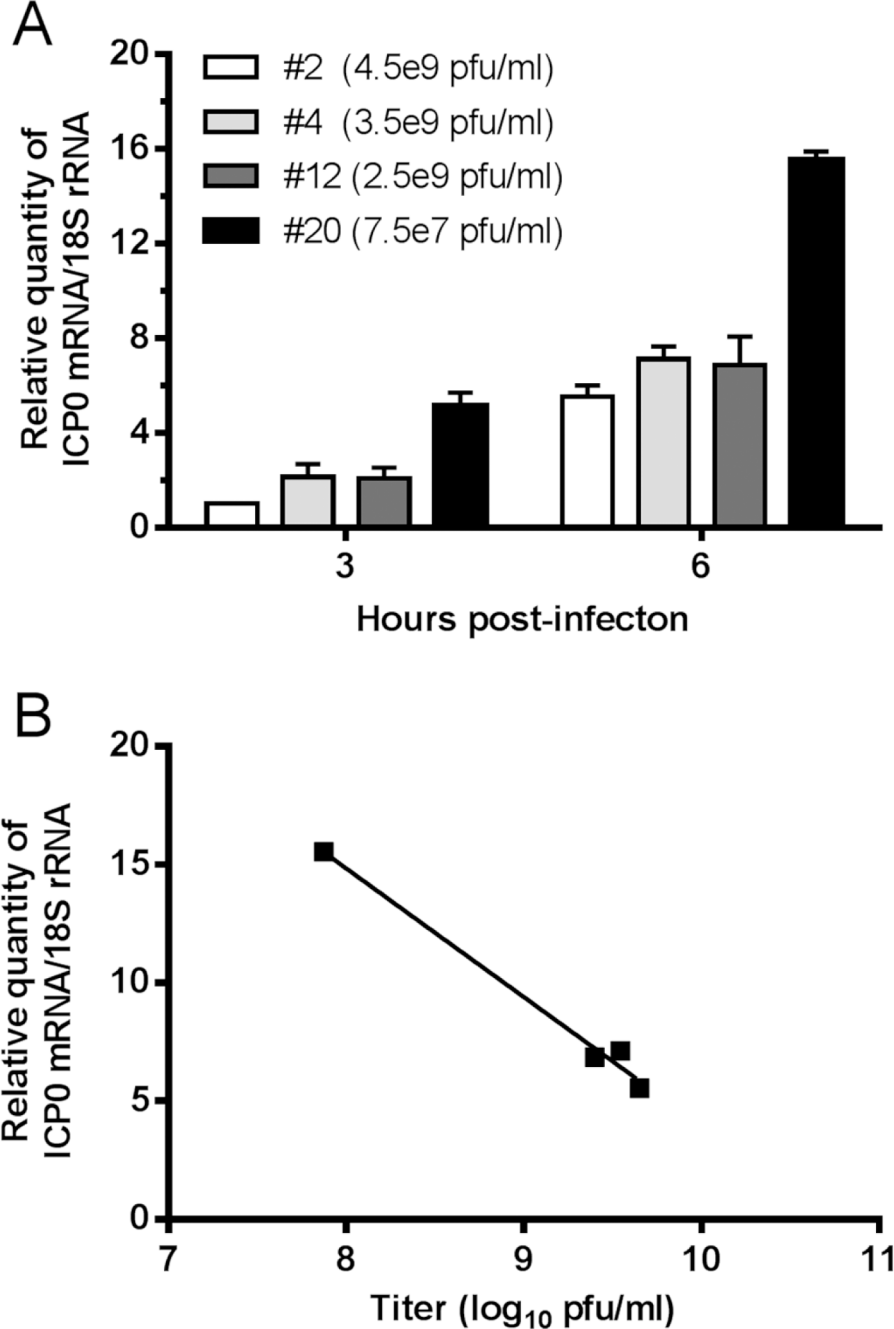
ICP0 transcript levels correlate with β-gal expression. ELVIS cell monolayers were infected with the indicated HSV-1 isolate at an MOI of 1, or were left uninfected. Total RNA was isolated at 3 h and 6 h post-infection and reverse transcribed. The cDNA was used in quantitative real-time PCR assays to detect ICP0 mRNA and 18S rRNA. The ICP0 signal was normalized to the 18S rRNA signal. A) The signal of clinical isolate #2 at 3 h post-infection was set to 1, and the relative amounts of ICP0 for the other isolates were calculated. cDNA from uninfected cells gave no signal. B) Correlation between the relative quantity of ICP0 signal and titer of virus in the stock.

The Herpevac Trial results underscored the importance of being able to measure and compare neutralizing activity against HSV-1 and HSV-2 and in individual subject’s serum. When we added equivalent pfu of different HSV-1 and HSV-2 isolates to ELVIS cells in the absence of test sera, we were surprised to find that the resulting OD readings varied considerably. Thus we needed to identify the cause underlying the difference in OD readings to standardize the assay for comparison of multiple virus isolates. First, we compared plaque formation on Vero cells, the cell line commonly used to determine virus titer, and on ELVIS cells. The differences in plaque forming efficiency of 5 isolates on Vero cells versus ELVIS cells were ≤3-fold, and did not consistently correlate with the difference in OD readings. Next, particle counts were determined by electron microscopy by comparison to latex beads of known concentration. Two extracellular virus stocks of an HSV-1 isolate differed almost 5-fold in infectious virus titer, but their particle to pfu ratios were each approximately 100:1 (data not shown). Thus, stock-to-stock variation in particle to pfu ratio was not an explanation for the differences in OD readings when the same amounts of infectious virus were added to ELVIS cells. The OD reading obtained from infected ELVIS cells depends upon transcriptional activation of the ICP6 promoter driving *lacZ* expression. This promoter responds primarily to ICP0 [20]. We therefore assessed the level of ICP0 transcript in cells infected with various HSV-1 isolates by quantitative real-time PCR. Four isolates were chosen: Stocks of isolates 2, 4, and 12 had similar titers (2.5 to 4.5x10^9^ pfu/ml); the fourth, isolate 20, had a much lower titer (7.5x10^7^ pfu/ml). Nonetheless, when diluted equivalently and added to ELVIS cells, all four viruses achieved equivalent OD readings after 45 min incubation with CPRG even though fewer pfu of isolate 20 had been used. To assess ICP0 expression, ELVIS cells were infected at an MOI of 1 with each of the isolates. Isolates 2, 4, or 12 produced similar levels of ICP0 transcript at 3 h post-infection and these levels had increased to approximately the same extent by 6 h post-infection (Fig 4A). In contrast, cells infected with isolate 20 at the same MOI contained much higher levels of ICP0 transcript at both time points. Thus, the ICP0 expression level for these virus isolates was inversely proportional to stock titer (Fig 4B), indicating that the level of ICP0 synthesized by a virus rather than the viral titer appears to correlate with β-gal expression in ELVIS cells.

### Optimizing assay read-out

**Fig 5:**
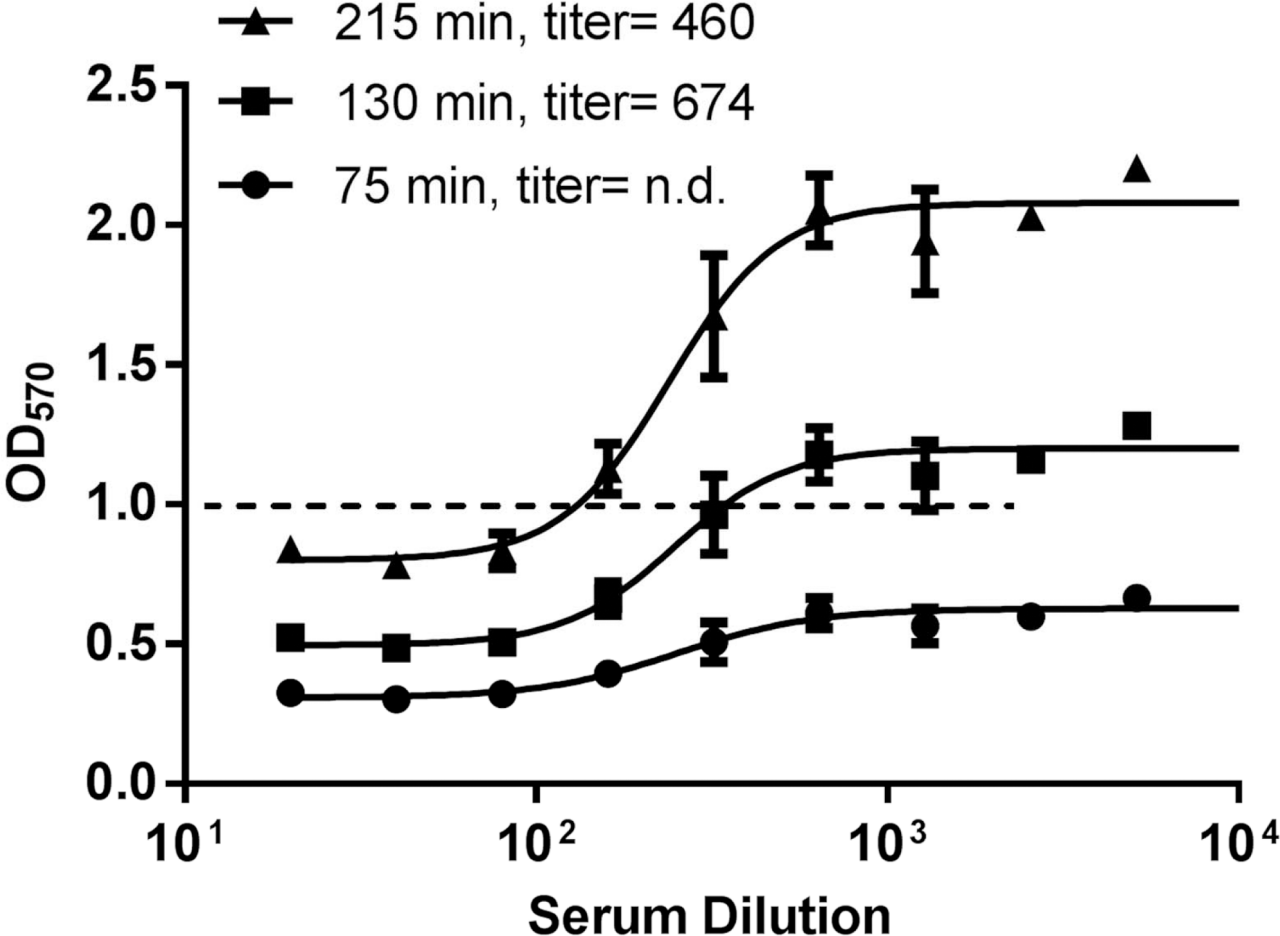
Comparison of CPRG substrate incubation times. A standard neutralization assay was set up as described in Fig 1. After thawing the frozen plate, CPRG was added, and then the plate was incubated for 75 min, 130 min, and 215 min before reading OD_570_. Data represent the mean ± SEM of duplicate samples. n.d., not determined.

Because of the apparent difference in level of β-gal expression induced by different virus isolates, we standardized the virus stocks by a different method using incubation time with substrate as the variable. Each virus stock was serially diluted and then transferred to ELVIS cell monolayers. Cells were lysed after 18 h of infection, plates were frozen and thawed, CPRG substrate was added, and OD_570_ readings were determined every 15 min. All viruses achieved an OD of 1.0 after varying lengths of incubation time with CPRG, and the lowest amount of virus that could be used such that all viruses eventually yielded an OD of 1.0 was 2.5x10^2^ pfu/well (not shown). Thus, 2.5x10^2^ pfu/well was chosen for assays comparing serum neutralizing capacity against various virus isolates. The incubation time determined for a given virus isolate could therefore be used in all subsequent assays with the isolate. Neutralizing antibody titers for a panel of test sera were above the limit of detection against all the viruses (not shown). An example of the effect of CPRG incubation time on OD readings and neutralizing antibody titer is shown in Fig 5.

**Fig 6:**
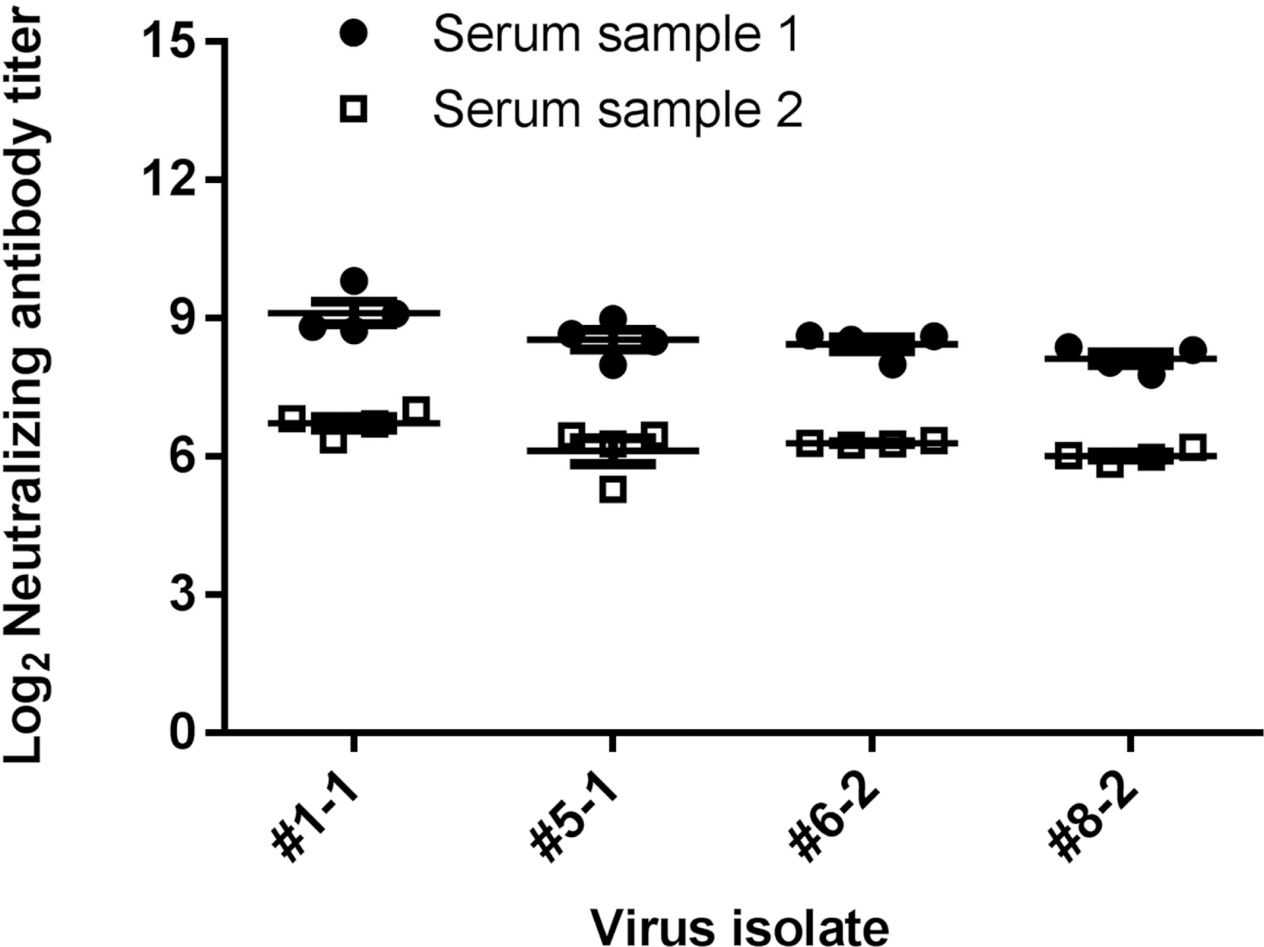
Neutralizing antibody titers obtained for individual sera are reproducible across assays. Post-vaccination sera from two different vaccinated test subjects that represented a low and intermediate response were tested against two HSV-1 primary isolates (#1 and 5) and two HSV-2 primary isolates (#6 and 8). Each symbol represents the titer derived in an independent neutralization assay. The mean ± SEM of 4 assays with each serum are shown.

The neutralization assay proved reproducible in two ways. First, neutralizing antibody titers of a single test serum against different stocks of the same virus isolate were equivalent (not shown). Reproducibility of the neutralization titer determination was also assessed by performing assays with the same virus isolate and sera over multiple days. Sera from two different subjects were selected which represented low and intermediate response patterns. Their neutralizing activity was assessed against HSV-1 and HSV-2 isolates in four independent assays performed on different dates (Fig 6). Reproducible neutralizing antibody titers were obtained against each virus, whether the serum had relatively low activity or more robust neutralizing capacity.

### Summary of neutralization assay throughput efficiency

After optimization of the several parameters discussed above, the assay can be performed in a minimum of 46 h, from the time cells are seeded into wells in the late afternoon of day 1 until data are acquired in the early afternoon of day 3. Overall, only about 3 h hands on time is required for sample/plate manipulation, depending on the number of plates prepared at once. Because very little technical time is required on day 1 and day 3 of the assay, assays may be staggered so that three assays are completed during a 5 d work week. In addition, several assays may be run independently and stored frozen until such time as is convenient to collectively thaw and develop them. Four serum samples can be run per plate against a virus isolate; thus, neutralization data for HSV-1 versus HSV-2 on 4 serum samples can be accomplished with just 2 plates, and we find that a technician can easily manipulate 10 plates per assay.

### Application: Neutralization of HSV-1 versus HSV-2 isolates

**Fig 7:**
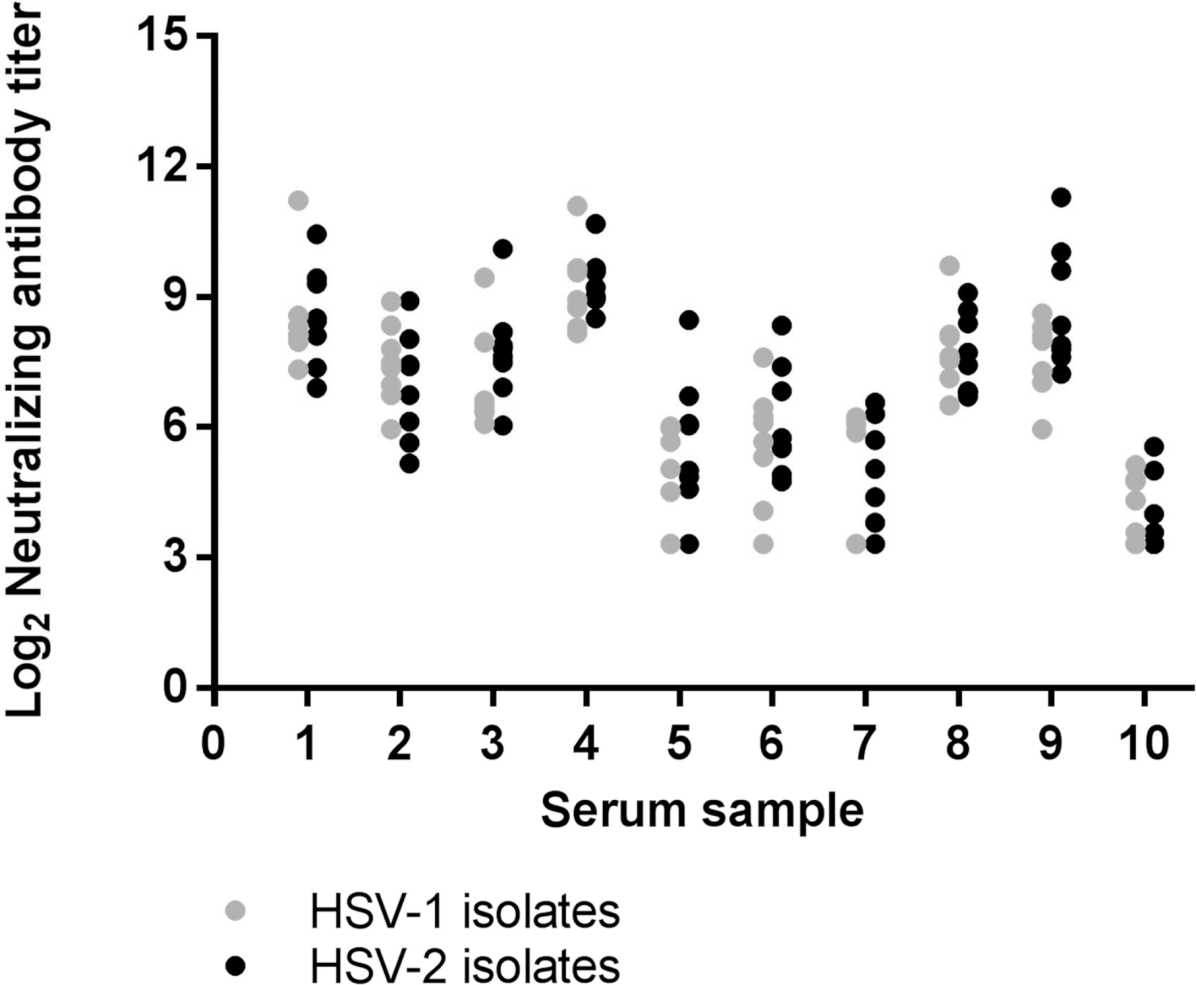
Sera from HSV-2 gD-vaccinated subjects neutralize HSV-1 and HSV-2 isolates with equal potency. Individual sera from 10 gD vaccine recipients were tested for neutralizing activity against 8 HSV-1 isolates (gray dots) and 8 HSV-2 isolates (black dots). P >0.05 (not significant) when comparing titers against HSV-1 and HSV-2 for each serum by paired t test.

We used the optimized neutralization assay to determine whether HSV-2 gD vaccine-immune sera had differential capacity to neutralize HSV-1 and HSV-2. Post-vaccination sera from 10 gD-vaccinated subjects were diluted and mixed with 8 HSV-1 primary isolates and 8 HSV-2 primary isolates. The 10 subjects’ sera showed a broad range of neutralizing activity, with 3 subjects, #1, 4 and 8, responding well to all virus isolates (Fig 7). Individual virus strains were neutralized to greater or lesser extents, but neutralizing titer against HSV-1 versus HSV-2 did not differ significantly (Fig 7). Thus, vaccination with HSV-2 gD typically stimulates antibody with the capacity to neutralize a variety of HSV-1 and HSV-2 wild-type circulating strains with equal efficiency.

## Discussion

The HSV neutralizing antibody method reported here has a consistent, objective colorimetric readout that provides reliable results within just 48 h. Our assay requires significantly less time than previous methods, provides objective readings of infectivity reduction, and has been optimized for use with any HSV-1 or HSV-2 strain. The higher throughput nature of this method will permit determination of neutralizing antibody titers in hundreds of serum samples, as would be obtained in a clinical trial. Differences in immunogenic properties of geographically disparate HSV isolates have been reported [21]. Thus, the ability to compare immune responses against a panel of clinical isolates also represents a distinct advantage of this method.

The total time required for a non-diagnostic assay is less important than the total amount of labor involved. This ELVIS cell-based assay requires a minimum of 46 h to complete, from the time cells are seeded into plates until the data is analyzed. Only about 3 h is required for sample/plate manipulation. Assays may be staggered so that many can be accomplished in overlapping fashion. Plates can also be stored frozen and thawed in large batches for data acquisition. Thus, although not truly high throughput in the sense of being fully automated, the assay has attractive features amenable to large clinical trials. The quantitative and objective nature of the assay and the ability to apply it in comparing multiple virus isolates provide additional benefits.

We optimized conditions for neutralization which included incubation time and the inclusion of complement. Complement functions as an important component of innate immune defense and is one of the earliest host responses to virus infection [22]. The usual method for detecting antiviral antibody involves heat-inactivation of serum in order to remove nonspecific inhibitors. Because this procedure reduces specific neutralizing activity which is recoverable with the addition of fresh nonimmune serum, it was concluded that neutralizing antibody, while itself heat stable, depends for its activity at least in part on the presence of complement [23]. Complement enhances the neutralizing activity of serum antibody from patients infected with HSV-1 or HSV-2 approximately 2 to 10-fold [23–26]. Complement has a similar effect on HSV-neutralizing activity of vaccinated mice and guinea pigs [3,27]. We also found that complement enhances neutralization for the majority of sera from subjects vaccinated with gD2. This is particularly true for neutralization of HSV-2 clinical isolates which was increased more than 5-fold on average, consistent with previous observations [25]. While HSV-1 neutralization improved by only 1.5-fold, a larger sample size might show a small positive effect of complement on neutralizing activity. The reasons for the inter-typic difference in effect of complement may relate to the masking of HSV-2 gD by other envelope glycoproteins to a greater extent than HSV-1 [4,5] and complement may be more important to the biological activity of antibody for neutralization of HSV-2.

HSV-1 and HSV-2 encode gC which helps protect the virion by binding to and inhibiting activation of complement component C3b [18,19]. gC reportedly binds human C3b more avidly than guinea pig C3b [28], implying that neutralizing antibody titers in the presence of human serum should be lower than with guinea pig serum. However, we found that guinea pig and human complement sources yielded similar neutralizing antibody titers. This result validates the use of guinea pig complement in our higher throughput assay. Our finding that neutralizing titers were higher against HSV-2 than HSV-1 in the presence of either human or guinea pig complement supports the notion that HSV-2 gC inhibits complement activation less potently than HSV-1 gC.

Addition of complement to the assay reported here may also explain the difference in conclusions reached in this study of sera from the Herpevac Trial and a previous study. Awasthi et al. [4] had detected 3.5-fold higher neutralization of HSV-1 than HSV-2 laboratory strains by sera from gD-vaccinated subjects in a plaque reduction assay in the absence of complement. Interestingly, when they analyzed neutralizing antibody responses to additional virus isolates, the differential between neutralization of HSV-1 and HSV-2 strains dropped to 2.3-fold [4], suggesting virus strain-dependent differences may also have influenced the differential in neutralization of HSV-1 versus HSV-2 they observed. Only slightly (1.5-fold) enhanced neutralization of HSV-1 occurred in our assays in the absence of complement. With the addition of complement, however, we found no consistent difference in capacity of sera from the Herpevac Trial to neutralize HSV-1 versus HSV-2 clinical isolates, due largely to the complement-aided increase in neutralization of HSV-2 isolates.

The assay described here utilized incubation times with substrate that were specific to each virus isolate. We searched for an explanation for the difference between pfu added and OD readout at a specified incubation time. Differential use of receptors or dependence on receptor density on Vero versus BHK cells was considered [29] but discarded when plaquing efficiency of various isolates on the two cell types did not consistently correlate with OD readings. Cell lysate virus stocks contain a significant amount of cellular debris, likely contributing to the poor reproducibility and lower neutralizing antibody titers obtained when using cell lysates compared with extracellular virus stocks. Particle to pfu ratios determined for two extracellular virus preparations of the same HSV-1 isolate were highly similar. Thus, a larger proportion of decoy or defective interfering particles were not an underlying reason for variation in OD readings at the same input pfu. One of the earliest events in HSV infection is expression of ICP0 [30]. We found that ICP0 transcript levels early after infection of ELVIS cells negatively correlate the amount of virus needed to achieve an OD reading of 1.0, likely explaining the difference between extracellular virus stocks when used at equivalent pfu. Comparison of ICP0 promoter sequences may yield an explanation for strain-to-strain variation in ICP0 expression levels.

Neutralizing antibody assays assess the functional blockade of virus infectivity. ELISA or RIA titers measured against HSV gD are consistently much higher because they measure antibodies that bind antigen in a non-neutralizing manner as well as antibodies that could neutralize [4–6,27,31]. However, a strong correlation exists between ELISA or RIA titers and neutralizing activity whether considering individual subject sera [4,31,32] or fold increase in peak titers of subject groups after successive vaccine doses [1,6]. Conclusions reached using ELISA occasionally are inconsistent with assays of neutralizing ability when comparing titers against gD1 versus gD2. One report analyzing sera from gD2-vaccinated subjects indicated no difference in titers by ELISA, but 2.3-fold lower neutralizing antibody titers against primary isolates of HSV-2 than HSV-1 in the absence of complement [4]. Various types of plaque reduction assays also differ in their sensitivity, with the 50% end point titer method yielding 10-fold higher neutralizing titers than the log_10_ method [2,4]. Greater sensitivity would be advantageous when serum neutralizing titers are low, such as for measurement of subject sera after first vaccine dose or monitoring decay of activity over time post-vaccination. It will be of interest to determine the degree of correspondence between the rapid assay method reported here and the 50% end point plaque reduction assay when comparing a panel of subject sera against a panel of primary clinical isolates.
